# Higher levels of cardiovascular fitness are associated with better executive function and prefrontal oxygenation in younger and older women

**DOI:** 10.3389/fnhum.2015.00066

**Published:** 2015-02-18

**Authors:** Olivier Dupuy, Claudine J. Gauthier, Sarah A. Fraser, Laurence Desjardins-Crèpeau, Michèle Desjardins, Said Mekary, Frederic Lesage, Rick D. Hoge, Philippe Pouliot, Louis Bherer

**Affiliations:** ^1^Centre PERFORM, Université ConcordiaMontreal, QC, Canada; ^2^Centre de Recherche de l'Institut de Gériatrie de MontréalMontreal, QC, Canada; ^3^Laboratoire MOVE (EA6314), Faculté des Sciences du Sport de Poitiers, Université de PoitiersPoitiers, France; ^4^Max Planck Institute for Human Cognitive and Brain SciencesLeipzig, Germany; ^5^Department of Social Work, McGill UniversityMontreal, QC, Canada; ^6^Departement de Physiologie, Université de MontréalMontreal, QC, Canada; ^7^École Polytechnique de MontréalMontreal, QC, Canada

**Keywords:** fitness, stroop, executive function, cerebral oxygenation, prefrontal cortex, right inferior frontal gyrus

## Abstract

**Aim**: Many studies have suggested that physical exercise training improves cognition and more selectively executive functions. There is a growing interest to clarify the neurophysiological mechanisms that underlie this effect. The aim of the current study was to evaluate the neurophysiological changes in cerebral oxygenation associated with physical fitness level and executive functions.

**Method:** In this study, 22 younger and 36 older women underwent a maximal graded continuous test (i.e., $\dot{\text{V}}$O_2*max*_) in order to classify them into a fitness group (higher vs. lower fit). All participants completed neuropsychological paper and pencil testing and a computerized Stroop task (which contained executive and non-executive conditions) in which the change in prefrontal cortex oxygenation was evaluated with near infrared spectroscopy (NIRS).

**Results:** Our findings revealed a Fitness × Condition interaction (*p* < 0.05) such that higher fit women scored better on measures of executive functions than lower fit women. In comparison to lower fit women, higher fit women had faster reaction times in the Executive condition of the computerized Stroop task. No significant effect was observed in the non-executive condition of the test and no interactions were found with age. In measures of cerebral oxygenation (ΔHbT and ΔHbO_2_), we found a main effect of fitness on cerebral oxygenation during the Stroop task such that only high fit women demonstrated a significant increase in the right inferior frontal gyrus.

**Discussion/Conclusion:** Higher fit individuals who demonstrate better cardiorespiratory functions (as measured by $\dot{\text{V}}$O_2*max*_) show faster reaction times and greater cerebral oxygenation in the right inferior frontal gyrus than women with lower fitness levels. The lack of interaction with age, suggests that good cardiorespiratory functions can have a positive impact on cognition, regardless of age.

## Introduction

Several studies have demonstrated that the aging process is accompanied by a decline in numerous cognitive domains (Drag and Bieliauskas, 2010; Salthouse, 2010). Among cognitive processes, executive functions and attentional control are particularly sensitive to aging (McDowd and Shaw, 2000). Executive functions generally refer to “higher-level” functions (planning, inhibiting, switching) involved in the control and regulation of cognitive processes (Miyake et al., 2000). Several neuroimaging studies have supported that executive functions such as inhibition and switching are under control of the frontal and prefrontal cortex (see reviews: Fuster, 2000; Turner and Spreng, 2012). The age-related deterioration of these abilities has often been associated with substantial anatomical and physiological changes (Bishop et al., 2010) and particularly in the frontal areas of the cerebral cortex (Yuki et al., 2012). However, it has also been suggested that aged-related brain atrophy and age-associated neurophysiological changes can be reduced with regular physical activity (Yuki et al., 2012).

Currently, it is recognized that regular physical activity is the best strategy (Deweerdt, 2011) to promote general health (physical and cognitive). Several studies have supported a delay in cognitive decline in physically active individuals (Angevaren et al., 2008). Several reviews on cardiovascular fitness and neurocognitive functions in older adults (Spirduso, 1980; McAuley et al., 2004) suggests “that maintaining higher levels of aerobic fitness may protect the brain against the normal effects of aging as well as cumulative effects of age-associated health problems” (McAuley et al., 2004, p. 218) but also proposes that more research is needed to clarify the underlying mechanisms of these changes in humans. Intervention studies also tend to support the notion that physical activity can reduce cognitive decline and lead to cognitive improvement in older adults. These studies suggest that aerobic fitness training is an effective way to enhance cognition in older adults and more selectively the executive functions of older adults, which are at particular risk for decline with age. Indeed, the results of the Colcombe and Kramer (2003) meta-analysis support improvements in several cognitive domains after aerobic training but these effects were greatest in the executive function domain. These results are further supported by current fitness level and intervention research (Boucard et al., 2012; Predovan et al., 2012). In their cross-sectional study, Boucard et al. (2012) demonstrated that physical fitness was selectively associated with better inhibitory control. Furthermore, Predovan et al. (2012) observed, that in comparison to wait-list controls, individuals who completed 3 months of aerobic training showed significant improvements in the Executive (inhibition/switch) condition of the Stroop task. In addition, only the trained group demonstrated a significant correlation between this executive measure and fitness level ($\dot{\text{V}}$O_2*max*_ estimate).

One shortcoming of many of the studies that examine the impact of physical fitness on cognitive performances is that physical fitness is often measured by self-reported questionnaires or submaximal tests. The types of questionnaires used are typically not adapted to older populations and are sensitive to social desirability, leading to an overestimation of the fitness level in older adults. Since older adults are often more sedentary than younger adults, this may increase the chance that a self-reported active older person would in fact be more sedentary than a self-reported sedentary younger adult. The gold standard index of physical fitness and cardiorespiratory health is $\dot{\text{V}}$O_2*max*_, which can be evaluated during a maximal graded exercise test using a computerized indirect calorimetric system. Physical fitness level calculated with the $\dot{\text{V}}$O_2*max*_ correlate with global cognitive function (Brown et al., 2010; Davenport et al., 2012; however see Etnier et al., 2006 for exception), which suggests that using $\dot{\text{V}}$O_2*max*_ is an important methodological choice when evaluating the associations between fitness level and cognitive function.

Given that there have been studies that have demonstrated a selective effect of fitness level on cognitive performance (Hall et al., 2001; Colcombe and Kramer, 2003; Bherer et al., 2013; Guiney and Machado, 2013) a growing body of literature has focused on understanding how physical training enhances cognitive performance. The preliminary research on this topic has fostered the development of theoretical models that assess and attempt to identify the neurophysiological pathways by which physical training enhances cognitive function. Several anatomical and neurophysiological parameters seem to be associated with improvement in executive performance in healthy fit subjects. Several studies demonstrate that regular physical training influences brain plasticity and increases the gray (Colcombe et al., 2003, 2004; Weinstein et al., 2012; Yuki et al., 2012) and white matter volume (Colcombe et al., 2003; Johnson et al., 2012; Tseng et al., 2013) in the brain, as well as an increase in the hippocampal volume (Erickson et al., 2009; Chaddock et al., 2010; Erickson et al., 2011; Szabo et al., 2011). Recently, Weinstein et al. (2012) reported that older subjects with a higher $\dot{\text{V}}$O_2*max*_ demonstrated better executive performance in the Stroop task and that this was associated with a greater prefrontal cortex volume. In line with animal studies on this topic, factors such as brain derived neurotrophic factors (BDNF) serve to promote neurogenesis, angiogenesis and synaptogenesis, most likely supporting the improvement in cognition (Cotman et al., 2007; Davenport et al., 2012). However, additional research is needed to support this hypothesis in humans.

Furthermore, other neurophysiological mechanisms could explain the relationship between cardiorespiratory fitness and cognitive function. Processing in the brain, including the cognitive processing is critically dependent on adequate blood flow to respond the energy and oxygen needs of the tissue, and this is mediated by the cerebral vasculature of the individual. Regular physical exercise is a powerful stimulus to improve vascular health and cerebral blood flow (Ainslie et al., 2008; DeVan and Seals, 2012). The positive relationship between cerebrovascular health and cardiorespiratory fitness is supported by evidence of angiogenesis and change in endothelial function (Bolduc et al., 2013), mediated by the liberation of BDNF and vascular growth factor (VEGF). Based on the preceding evidence this positive relationship between cardiorespiratory health and cognition could be mediated by vascular mechanisms (Davenport et al., 2012), such as cerebrovascular reserve, improving oxygen transport and delivery to the cerebral cortex.

Cerebral oxygenation seems to play an important role in regulation of cognitive processes. The availability of oxygen content in the brain seems to regulate positively or negatively cognitive processing. For Example, during hypoxia, when there is a lack of oxygen available to the brain, cognitive performance is poorer (Lieberman et al., 1994) and after glucose supplementation, which improves the cerebral oxygenation, cognitive performance is enhanced (Gagnon et al., 2012). In normal aging, in the absence of major neurological events, it's well known that aging is accompanied by a lower baseline cerebral blood flow (Bangen et al., 2009) and lower cerebral oxygenation (Mehagnoul-Schipper et al., 2002; Fisher et al., 2013). From a clinical standpoint, reduced cerebrovascular health can be linked to cognitive decline in older adults. During the aging process, the hypoperfusion (decrease in perfusion) can lead to cognitive impairment and increase the risk of developing neurological disorders and stroke (Bangen et al., 2009). Based on this evidence, it seems plausible that poor cardiovascular health and reduced cerebrovascular function may result in poor cognitive performance. Emerging neuroimaging technologies such as near infra-red spectroscopy (NIRS-optical imaging) provide a good measure of cerebrovascular health in aging adults (Suhr and Chelberg, 2013) and permit further investigation of the role of cerebral oxygenation in cognitive processing of higher fit individuals. Indeed, optical imaging is a promising tool to study neurovascular coupling and could be used to complement existing cognitive neuroscience data (Fabiani et al., 2014).

Although several animal studies have shown that physical training can improve angiogenesis and enhance the O_2_ transport in the brain (see reviews Davenport et al., 2012, p. 157; Bolduc et al., 2013) there are a limited number of studies that have evaluated the cerebral oxygenation in higher fit people. To the best of our knowledge, only two studies showed a positive effect of physical fitness on cerebral oxygenation. In a sample of patients with heart failure, Fu et al. (2013) observed an improvement on cerebral oxygenation during maximal exercise after an aerobic training program. Furthermore, Fabiani et al. (2014), observed a greater response in cerebral oxygenation during a visual task in higher fit people than sedentary control older subjects. To date, no study has evaluated the association between of physical fitness and cerebral oxygenation during a cognitive task in younger and older adults.

Taken together, existing evidence suggests a positive relationship between high levels of fitness and high levels of executive performance that may be related to cerebral oxygenation. However, to our knowledge, no study has examined all three of these factors in a sample of fit younger and older adults. Therefore, the current study was designed to assess fitness level, cerebral oxygenation changes and cognitive performances in young and old people, in order to explore if fitness level is associated with executive control in a Stroop task. In addition, we separated our sample into younger and older adults in order to explore if increasing age influenced the relationship between fitness level, executive control, and cerebral oxygenation. We hypothetized that within our sample, those that were more physically fit would perform better on the Stroop task and demonstrate better performances in the executive condition of the Stroop task, than individuals with lower physical fitness levels and that this difference would be related to increased cerebral oxygenation during the task in the higher fit individuals in comparison to the lower fit.

## Method

### Participants

Twenty-two young (age: 24.6 ± 3.6, range [19–34]) women and 36 old women (age: 62.9 ± 5.4, range [55–72]) participated in this study. All participants signed a written statement of informed consent. They were non-smokers, did not undergo major surgery 6 months prior to the experiment, did not report any neurological or psychiatric disorders and were not taking medication known to affect cognition. To exclude individuals with signs of dementia or depression, older participants completed the Mini-Mental State Examination (scores ranged between 26 and 30, Folstein et al., 1975), and the Geriatric Depression Scale (scores above 11 excluded). The younger participants completed the Beck Depression Inventory. Participants were screened for perceptual impairment by completing a questionnaire on auditory and visual function. Moreover, given the physical implications of the study, participants were also screened and excluded for cardiovascular disease, vascular peripheral attacks and moderate to severe hypertension based on self-report. The protocol was reviewed and approved by the Research Ethics Board of the Research Center of the Geriatrics Institute of Montreal (Canada), and has been conducted in accordance with recognized ethical standards and national/international laws.

### Study design

All participants completed a cardiorespiratory and a cognitive assessment over a 3-week period. During the first session participants signed the consent form and completed questionnaires on health and mental status. During the second session, participants completed the aerobic test and the clinical neuropsychological tests. Cerebral oxygenation was measured during the computerized Stroop task in a third session.

### Aerobic fitness assessment

The maximal continuous graded exercise test was performed on cycle ergometer (Lode). Oxygen uptake ($\dot{\text{V}}$O_2_) was determined continuously on a 15-s basis using an automated cardiopulmonary exercise system (Moxus, AEI Technologies, Naperville, IL, USA). Gas analyzers (S3A and CD3A, AEI Technologies, Naperville, IL, USA) were calibrated before each test, using a gas mixture of known concentration (15% O_2_ and 5% CO_2_) and ambient air. Their accuracy was ± 0.003% for oxygen and ± 0.02% for carbon dioxide (data provided by the manufacturer). The turbine was calibrated before each test using a motorized syringe (Vacu-Med, Ventura, CA, USA) with an accuracy of ± 1%. The tidal volume was set at 3 l.

Initial workload was set at 0.75 or 1 W per kilogram (kg) of body weight according to the participant's physical activity level and increased by 15 W every minute until voluntary exhaustion. Strong verbal encouragement was given throughout the test. The highest $\dot{\text{V}}$O_2_ over a 15-s period during the test was considered as maximal oxygen consumption ($\dot{\text{V}}$O_2*max*_, in ml.kg^−1^. min^−1^). To ensure patient security, electro-cardiographic activity was monitored continuously using a 12-lead ECG (Marquette, Missouri) and blood pressure was measured manually every 2 min using a sphygmomanometer. According recommendations made by Duncan et al. (1997) and Midgley et al. (2007), $\dot{\text{V}}$O_2*max*_ was defined when two of three criteria were satisfied: (1) a plateau in $\dot{\text{V}}$O_2_, (2) a heart rate > 90% or equivalent to their age predicted maximum (i.e., 220—age) and (3) a respiratory exchange ratio >1.1. Aerobic fitness group assignments (i.e., higher fit and lower fit) were based on age- and gender-referenced $\dot{\text{V}}$O_2*max*_ norms (Shvartz and Reibold, 1990). Subjects were considered “higher fit” when their $\dot{\text{V}}$O_2*max*_ correspond to the fitness level of categories 1-3 [excellent to good], and “lower fit” in categories 4–7 [average to very poor] (Labelle et al., 2013).

### Cognitive assessment

#### Neuropsychological tests

Psychomotor speed was measured with the Digit Symbol Substitution Test (DSST, WAIS-III, Wechsler, 1997). In this test, the participant had to associate symbols to numbers (1–9) by referring to a response key consisting of rectangles containing a number in the top part and a symbol in the bottom part. The participant had 120 s to draw as many symbols as possible.

The Trail Making test and the Modified Stroop color test (Bohnen et al., 1992, adapted in French) were used to assess attention and executive functions. In the Trail Making Test part A, the participant had to draw a line joining numbers (from 1 to 25) as fast as possible. In Part B, the participant had to draw a line alternating between letters in alphabetical order and numbers in ascending order (1-A-2-B-3-C, etc.) as fast as possible. The dependent variables are the time to complete each part. The paper and pencil, Modified Stroop color test includes four conditions and provides a measure of inhibition and mental flexibility. In the reading condition, the participant had to read aloud color words as fast as possible. In the naming condition she had to name the color of rectangles. In the interference condition, color-words were printed in a color that differs from their meaning (e.g., red printed in green) and the task was to name the color of the word (green in the example) and avoid reading the word. In the flexibility condition, the participant had to alternate between naming the color of the color-words, and reading the words (when the color-words appear in a square). In all conditions, words list were printed on a sheet of paper and participants had to provide their answer verbally as fast as possible. Dependent variables were the time to complete each condition and the number of errors committed (%).

#### Computerized stroop task

**Figure 1:**
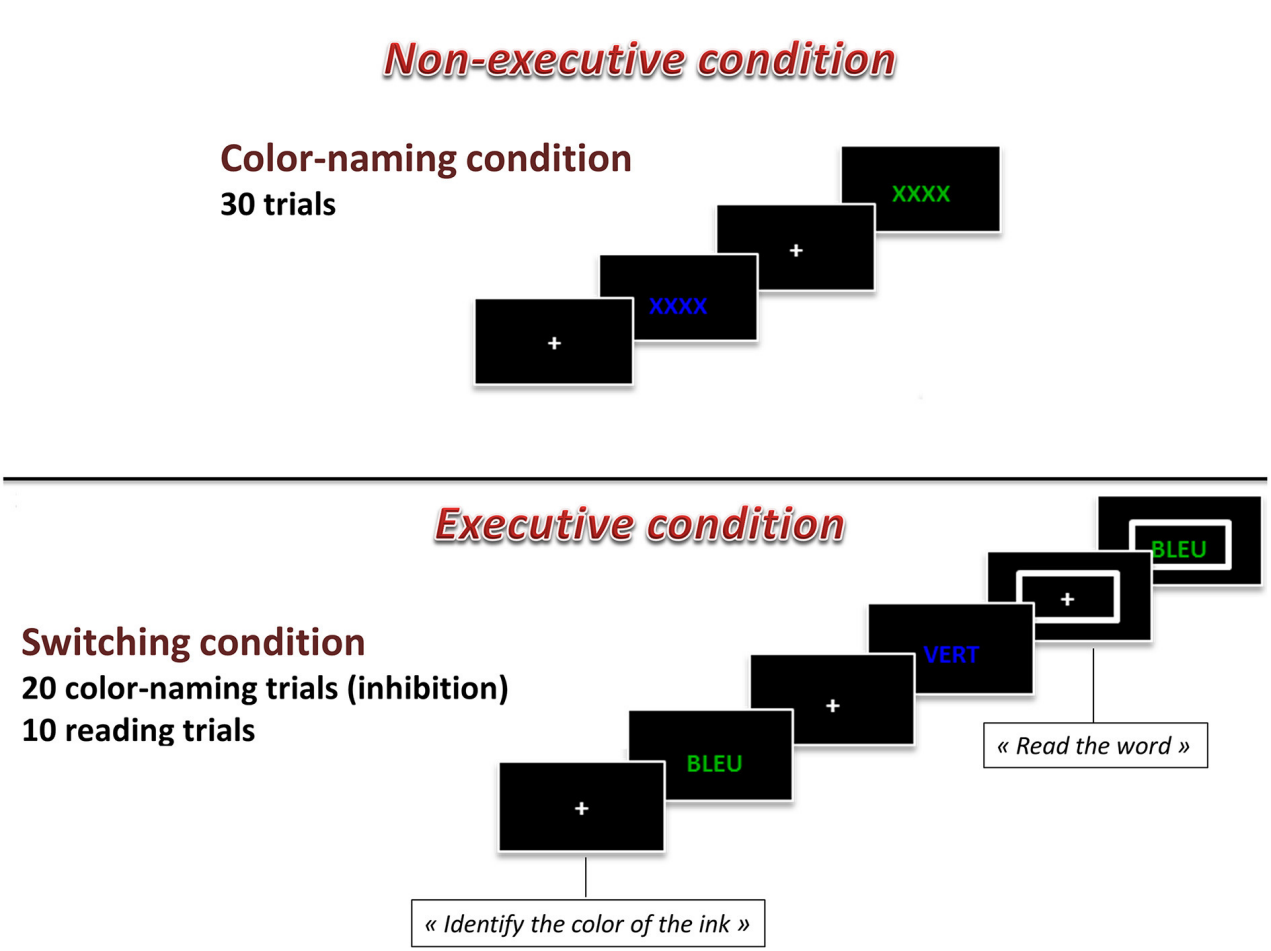
**Graphical representation of the computerized Stroop task**.

The Computerized Modified Stroop task was based on the Modified Stroop Color Test (Bohnen et al., 1992) and included two experimental conditions: Naming and Executive. Each block lasted 60 s, and was interspersed with 60-s resting blocks. Overall, there were four experimental task blocks (2 naming and 2 executive) and 5 resting blocks, for a total length of 9 min. All trials began with a fixation cross for 1.5 s, and all visual stimuli appeared in the center of the computer screen for 2.5 s. Participants provided their responses with two fingers of their right hand on a QWERTY keyboard. In the Naming block, participants were presented with a visual stimulus (XXXX) colored in green or in blue and participants were asked to identify the color of the ink with a button press. In the Executive block, each stimulus consisted of a color-word (BLUE or GREEN) printed in the incongruent ink color (i.e., the word BLUE was presented in green ink). Participants were asked to identify the color of the ink (i.e., green). In one third of the trials of the Executive block, a large rectangle appeared around the word. When this occurred, participants were instructed to read the word instead of identify the color of the ink (i.e., BLUE). As such, within the Executive block, there were both inhibition trials in which the participant had to inhibit their reading of the word and correctly identify the color of the ink and there were switch trials in which the participant had to switch their response mode to reading the word and not identifying the color of the ink when a rectangle appeared around the word presented. In total, there were 30 Naming trials and 30 Executive trials (20 inhibition and 10 switch trials). These two blocks of the computerized Stroop task are presented in the Figure 1. A practice session was completed before the acquisition run. The practice consisted of a shorter version of the task and included visual feedback when participants produced incorrect responses. Dependent variables were reaction times and the number of errors committed (%).

### Cerebral oxygenation

**Figure 2:**
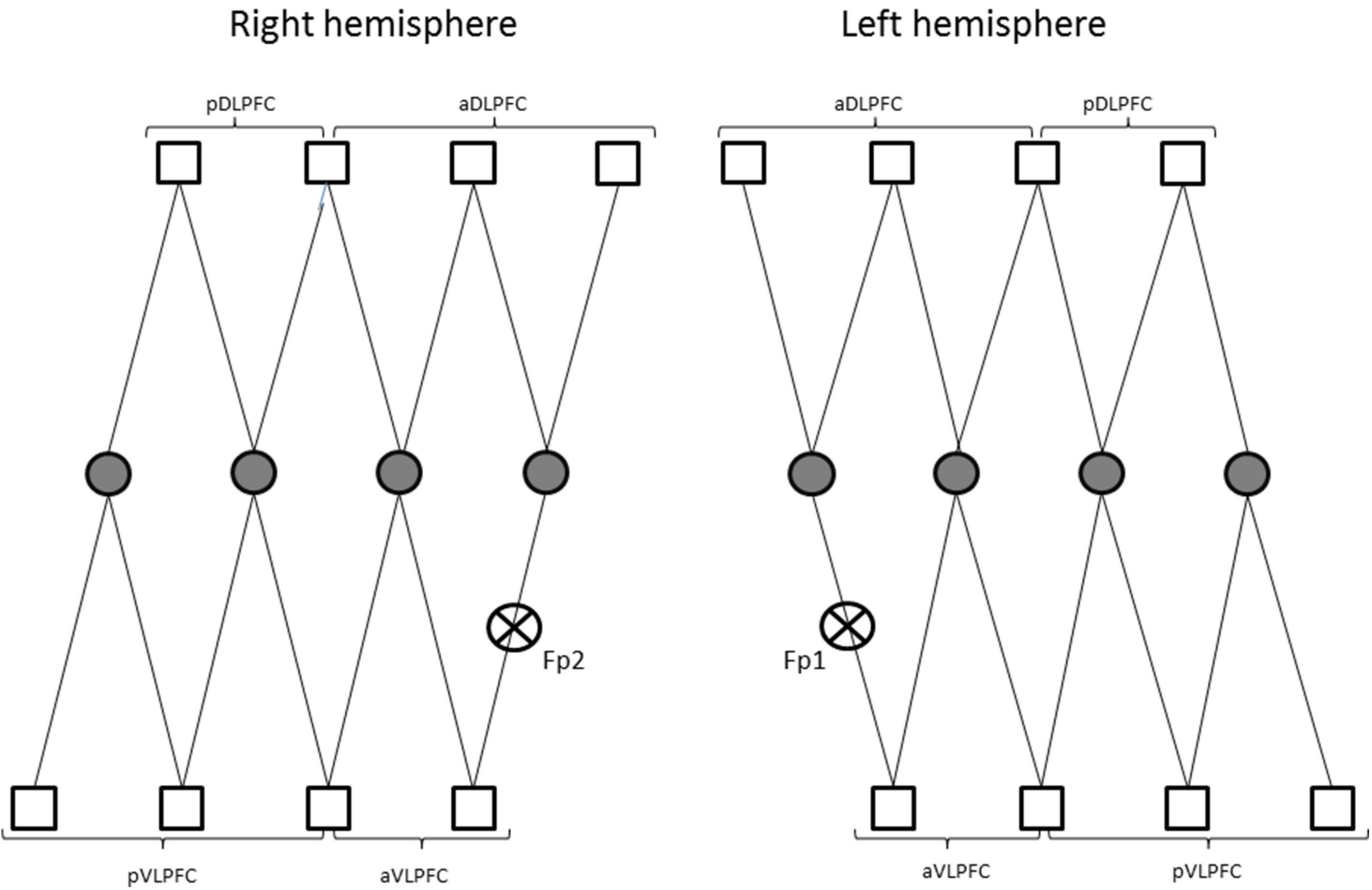
**Representations of the two arrays of 4 sources (circle) and 8 detectors (square) on plastic helmets covering prefrontal regions**. (pDLPFC, posterior dorsolateral prefrontal cortex; aDLPFC, anterior dorsolateral prefrontal; pVLPFC, posterior ventrolateral prefrontal cortex; aVLPFC, anterior ventrolateral prefrontal cortex; Fp1/Fp2, frontal mark according the 10/20 system; see Okamoto et al., 2004).

**Figure 3:**
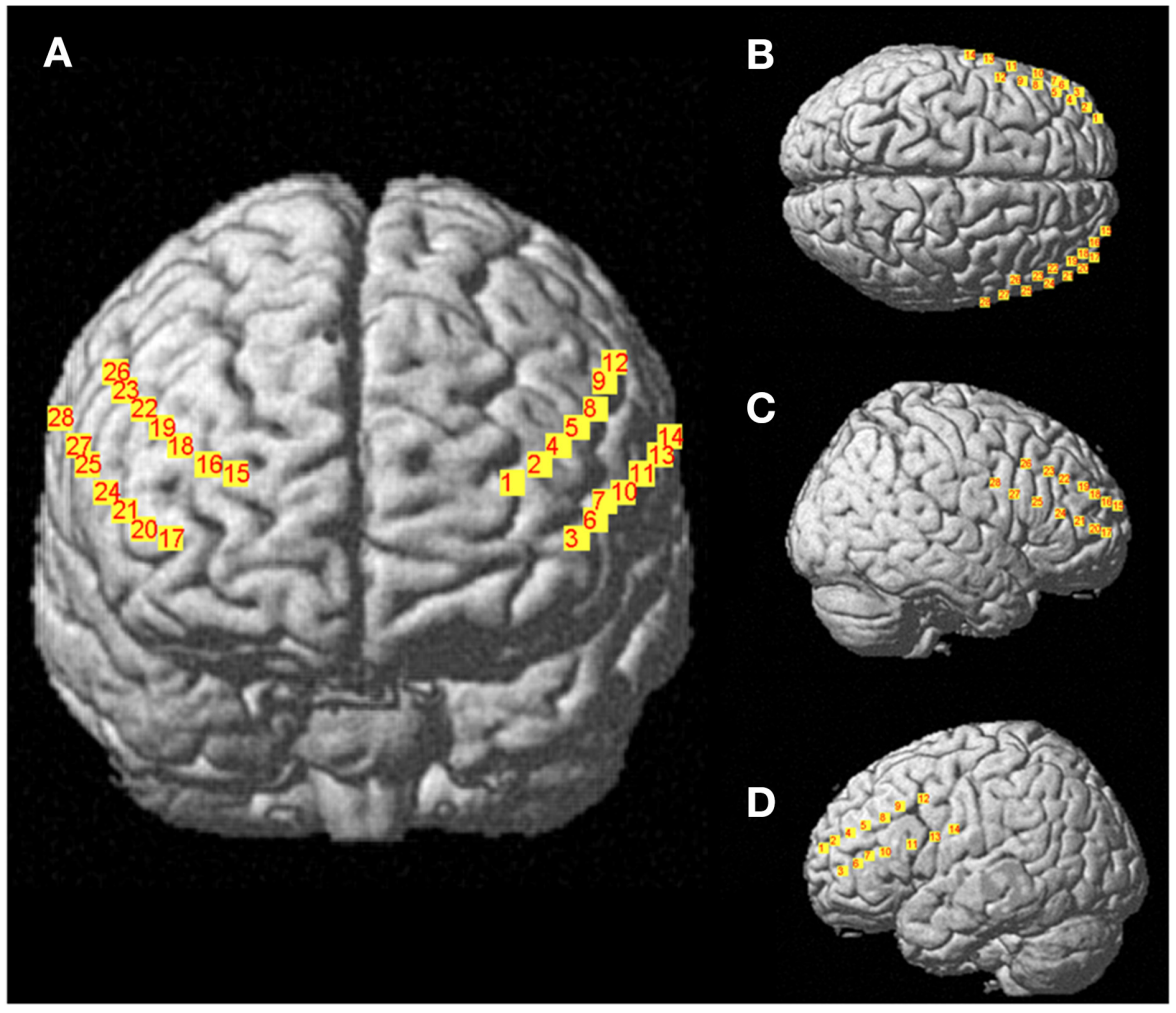
**Representations of 28 channels NIRS (i.e., detectors- sources) covering prefrontal regions (A), Frontal view; (B), Top view; (C), right sagittal view; (D), left sagittal view**.

Changes in concentration of oxyhemoglobin (HbO_2_) and deoxyhemoglobin (HbR) were measured by a multichannel, continuous-wave spectrometer (CW6, TechEn Inc., Milford, MA), using an 830 nm wavelength that is more sensitive to HbO_2_ and a 690 nm more sensitive to HbR. Combining the multispectral measurements with known extinction coefficients of hemoglobin, concentration changes of HbO_2_ was calculated using the modified Beer-Lambert and a path-length factor of 5.93. As illustrated in Figure 2, two arrays of 4 sources and 8 detectors were mounted on plastic helmets covering prefrontal regions. Two probes (one on each hemisphere) were arranged with one central, anterior-posterior row of four emitters per hemisphere. Sixteen detectors were placed strategically 2.8 cm away from the emitters, eight of them were dorsal, while the other eight were ventral, so that each probe had four dorsal detectors and for ventral detectors. The two probes were placed symmetrically over the lateral prefrontal cortex and the most anterior and most ventral pair of emitter-detector of each probe was placed on Fp1/Fp2 using the 10/20 system. Fp1 and Fp2 regions have been found to correspond to the superior and medial frontal gyri (Okamoto et al., 2004). This method and setup has already been used successfully in our previous research projects (Gagnon et al., 2012; Lague-Beauvais et al., 2012). The 14 source–detector pairs were combined into four different approximate regions of interests (ROI) that do not refer exactly to the underlying brain regions. They consisted of pairs 1–4 for the anterior DLPFC (BAs 9, 10 and 46), 5–7 for the posterior DLPFC (BAs 6 and 4), 8–10 for the anterior VLPFC (BAs 10, 45 and 46) and 11–14 for the posterior VLPFC (BAs 4, 6, and 44) for both hemispheres (Figure 1). The illustration of the brain mapping imaging is represented in Figure 3. The NIRS transmitters were tightly secured with a tensor bandage wrapped around the forehead, taking sufficient care to ensure that there was no interference of background light and to limit movement during cognitive task. Variables of interest were relative changes in concentration of ΔHbO_2_, ΔHbr, and ΔHbT compared to the baseline (1 min at rest before the computerized Stroop task) (Gagnon et al., 2012; Lague-Beauvais et al., 2012), because continuous-wave technology does not allow quantifying absolute concentration due to the incapacity of measuring optical path lengths (Delpy and Cope, 1997; Hoshi, 2003; Ferrari and Quaresima, 2012).

### Analysis of NIRS signal

NIRS data analysis was performed in Matlab (The MathWorks, Natick, MA) using the nirs10 toolbox (Pouliot et al., 2012) based on SPM and NIRS_SPM (Ye et al., 2009) with additional modules for ANOVAs. A principal component analysis was done to remove the 6 largest components from the data (Peng et al., 2014). A Butterworth order 2 high pass filter at 0.0042 Hz and a low pass filter with the shape of the SPM8 canonical hemodynamic response function (HRF) were applied. The analysis of NIRS signal was performed by block (i.e., by condition, including all trials), without separating out errors trials. A general linear model (GLM) was estimated for each NIRS channel and for each patient using this same HRF for each chromophore (HbO_2_, HbR, and HbT). The results were interpolated onto left, right and frontal 2-dimensional maps (Ye et al., 2009), assuming identical NIRS source and detector positions for all subjects.

### Statistical analyses

Standard statistical methods were used for the calculation of means and standard deviations. Normal Gaussian distribution of the data was verified by the Shapiro–Wilks test and homoscedasticity by a modified Levene Test. The compound symmetry, or sphericity, was checked by the Mauchley test. When the assumption of sphericity was not met, the significance of F-ratios was adjusted according to the Greenhouse–Geisser procedure when the epsilon correction factor was <0.75, or according to the Huyn–Feld procedure when the Epsilon correction factor was >0.75. An analysis of variance (Age x Fitness x Measures) was performed. For the NIRS data, a mixed Two-Way ANOVA (fitness by condition) was estimated pixel wise on the interpolated maps and corrected for multiple comparisons using the Euler characteristic methodology (Li et al., 2012). For Trail test, Stroop test (paper pencil and computerized version), we performed an ANOVA (Age × Fitness × Condition). A *post-hoc* multiple comparisons Bonferroni test was performed. The magnitude of the difference was assessed by the Effect Size (ES), as presented elsewhere (Dupuy et al., 2010). The magnitude of the difference was considered either small (0.2 < ES < 0.5), moderate (0.5 < ES < 0.8), or large (ES > 0.8) (Cohen, 1988). The significance level was set at *p* < 0.05 for all analyses.

## Results

**Figure 4:**
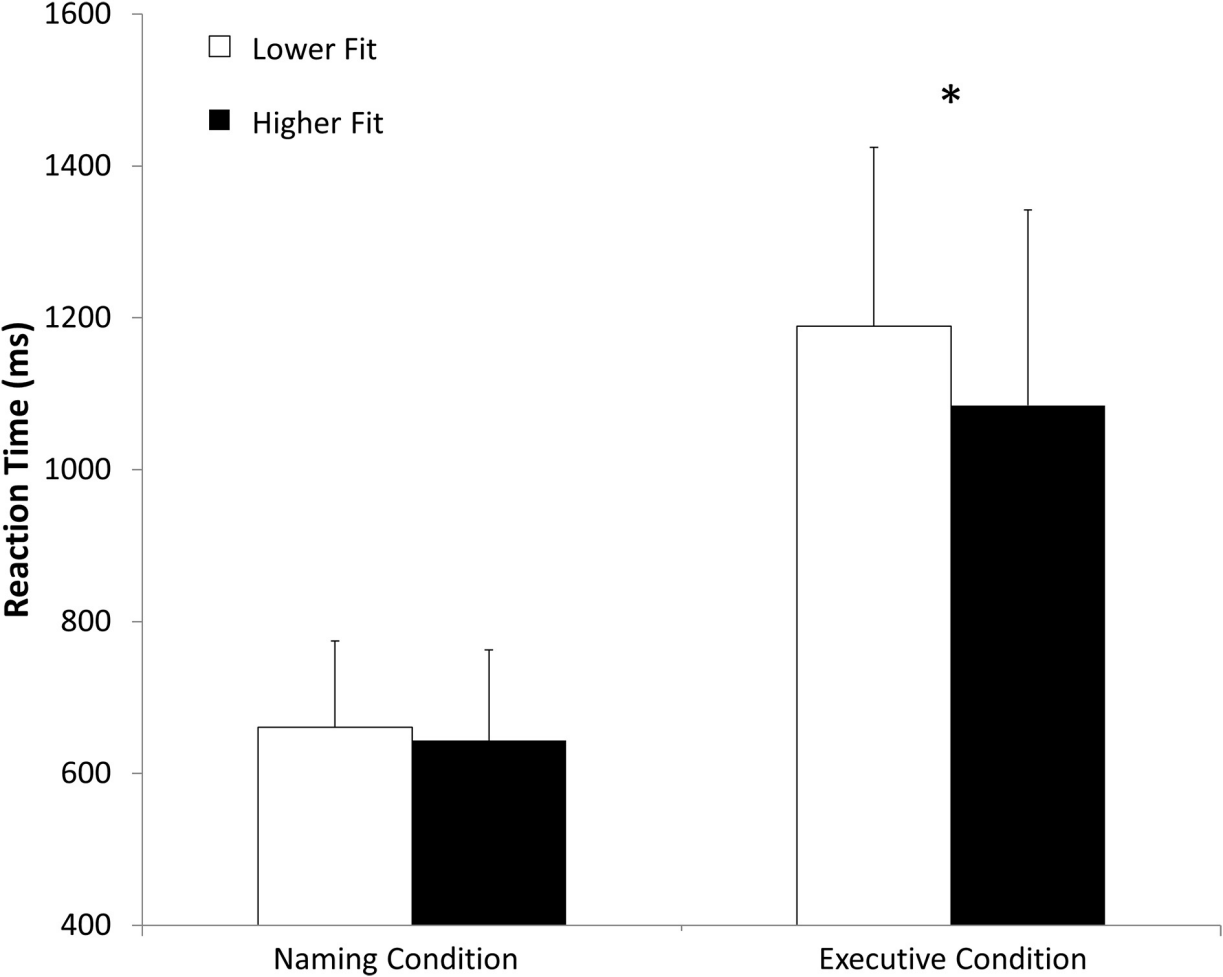
Mean reaction time (ms) in naming and executive conditions for higher fit and lower fit women. ^*^p < 0.05.

Concerning the paper and pencil neuropsychological tests, the ANOVA showed a main effect of age on the substitution test [*F*_(3, 54)_ = 30.06, *p* < 0.01], on the similarities test *F*_(3, 54)_ = 4.50, *p* < 0.05, on the Stroop task [*F*_(1, 54)_ = 8.57, *p* < 0.01] and on the Trail Making test [*F*_(1, 54)_ = 15.96, *p* < 0.01]. We found also a condition × age interaction for the Stroop task [*F*_(1, 54)_ = 4.78, *p* < 0.05] and for the Trail Making test [*F*_(1, 54)_ = 8.41, *p* < 0.01]. We also found a main effect of age [*F*_(1, 54)_ = 53.0, *p* < 0.001] and fitness [*F*_(1, 54)_ = 19.0, *p* < 0.001] on $\dot{\text{V}}$O_2*max*_. All these results are presented in Table 1. There was no main effect of fitness on any of the paper and pencil neuropsychological tests. Concerning the reaction times in the computerized Stroop task, the ANOVA revealed a main effect of condition [*F*_(1, 54)_ = 342.8, *p* < 0.05], a Condition x Fitness interaction [*F*_(1, 54)_ = 5.6, *p* < 0.05] and a Condition × Age interaction [*F*_(1, 54)_ = 13.7, *p* < 0.05]. The condition × Fitness × Age interaction was non-significant. These results are presented in Figure 4 and Table 2. There was no main effect of fitness or age difference in the number of errors produced.

**Table 1:** **Means and standard deviations for the neuropsychological, mood and aerobic fitness assessment data in higher and lower fit participants**.

	**Younger**	**Older**		**Younger**			**Older**		
									
	**Total**	**Total**	**Age**	**Higher fit**	**Lower fit**	**Fitness**	**Higher fit**	**Lower fit**	**Fitness**
			**ES (d)**			**ES (d)**			**ES (d)**
Age	24.6 ± 3.6	62.9 ± 5.4	–	24.5 ± 3.1	23.5 ± 5.3	–	63.0 ± 3.1	60.8 ± 5.6	–
Education (years)	17.09 ± 1.9	15.7 ± 3.7	–	17.69 ± 1.7	15.5 ± 1.38	–	15.69 ± 3.1	15.5 ± 5.1	–
BDI/GDS (/30)	1.9 ± 2.0	3.5 ± 4.4	0.4	2.5 ± 2.8	1.7 ± 1.7	0.3	1.7 ± 1.6	3.9 ± 4.8	0.6
MMSE (/30)	–	28.67 ± 1.0	–	–	–	–	28.17 ± 1.0	28.77 ± 1.2	0.5
**SHORT TERM AND WORKING MEMORY**									
Forward span	9.9 ± 2.5	10.0 ± 2.5	0.0	10.2 ± 2.9	9.17 ± 1.2	0.5	10.3 ± 2.4	8.5 ± 1.6	0.8
Backward span	6.9 ± 2.6	6.9 ± 2.1	0.0	7.3 ± 2.4	6.0 ± 2.9	0.5	7.1 ± 2.2	5.8 ± 1.2	0.7
**ATTENTION AND PROCESSING SPEED**									
Similarities	26.6 ± 4.8	23.9 ± 4.0**^#^**	0.6	26.9 ± 5.1	25.8 ± 4.5	0.2	24.5 ± 3.8	21.1 ± 4.5	0.8
Substitution	90.5 ± 12.1	69.7 ± 11.6**^#^**	1.7	83.8 ± 12.4	93.1 ± 9.9	0.8	69.2 ± 12.2	72 ± 8.4	0.2
**PERCEPTUAL ABILITIES AND PROCESSING SPEED**									
Trail A (s)	25.9 ± 8.8	35.0 ± 10.5**^#^**	0.9	25.5 ± 8.8	27.1 ± 9.7	0.2	35.3 ± 11.2	33.6 ± 6.0	0.2
Stroop—reading (s)	19.6 ± 2.7	20.2 ± 3.8	0.2	19.5 ± 2.6	19.7 ± 3.3	0.1	19.8 ± 4.0	22.6 ± 1.7	1.0
Stroop—color naming (s)	26.2 ± 4.5	29.2 ± 5.5	0.6	25.7 ± 3.9	27.7 ± 6.1	0.4	28.2 ± 5.3	34.0 ± 4.0	1.2
**COGNITIVE INHIBITION AND FLEXIBILITY**									
Trail B (s)	53.8 ± 17.8	77.2 ± 21.3**^#^**	1.2	51.9 ± 15.0	58.8 ± 24.8	0.3	75.6 ± 19.7	85.2 ± 28.7	0.4
Stroop—inhibition (s)	43.5 ± 9.2	56.1 ± 13.4**^#^**	1.1	41.9 ± 7.0	47.6 ± 13.5	0.5	54.4 ± 12.4	64.5 ± 16.0	0.7
Stroop—switching (s)	50.9 ± 13.6	56.5 ± 15.0	0.4	49.5 ± 15.1	54.7 ± 8.1	0.4	54.8 ± 15.1	64.4 ± 11.0	0.7
**AEROBIC FITNESS**									
MAQ	4.9 ± 2.1	4.0 ± 2.9	0.3	5.6 ± 1.7	4.2 ± 2.5	0.6	3.9 ± 2.4	4.2 ± 3.4	0.1
$\dot{\text{V}}$O_2*max*_(ml.min^−1^.kg^−1^)	43.8 ± 8.0	28.7 ± 7.3**^#^**	1.9	46.6 ± 7.0	36.4 ± 5.3^*^	1.6	30.1 ± 1.5	21.4 ± 7.1^*^	1.7

*Results are presented mean ± SD*.

# *Different from younger p < 0.05*.

* *Different from high fit p < 0.05; ES (d), Cohen's d (Effect Size); MMSE, Mini Mental State Examination; BDI, Beck Depression Inventory; GDS, Geriatric Depression Scale; MAQ, Modifiable Activity Questionnaire; $\dot{\text{V}}$O_2*max*_, Maximal Oxygen Uptake*.

**Table 2:** **Means and Standard deviations of reaction times and accuracy (%) during both conditions of the Computerized Stroop task**.

	**Younger**	**Older**		**Younger**			**Older**		
									
	**Total**	**Total**	**Age**	**Lower fit**	**Higher fit**	**Fitness**	**Lower fit**	**Higher fit**	**Fitness**
			**ES (d)**			**ES (d)**			**ES (d)**
**REACTION TIME (MS)**									
Naming condition (ms)	567.1 ± 109.7	695.7 ± 94.4**^#^**	1.2	642.4 ± 146.9	538.9 ± 80.5	0.8	679.3 ± 78.3	699.0 ± 98.2	0.2
Executive condition (ms)	907.7 ± 224.8	1227.7 ± 185.7**^#^**	1.5	1091.5 ± 237.3	838.9 ± 182.7^*^	1.2	1286.5 ± 206.8	1215.9 ± 185.0^*^	0.4
**ACCURACY (%)**									
Naming condition (%)	99.4 ± 1.2	99.0 ± 1.9	0.2	99.5 ± 1.2	99.4 ± 1.2	0.1	99.5 ± 1.2	98.9 ± 2.0	0.3
Executive condition (%)	94.9 ± 4.5	89.2 ± 9.4	0.7	90.5 ± 2.9	96.5 ± 3.9	1.7	88.8 ± 3.9	89.3 ± 10.2	0.1

*Results are presented mean ± SD*.

# *Different from younger p < 0.05*.

* *Different from high fit p < 0.05; ES (d), Cohen's d (Effect Size)*.

**Figure 5:**
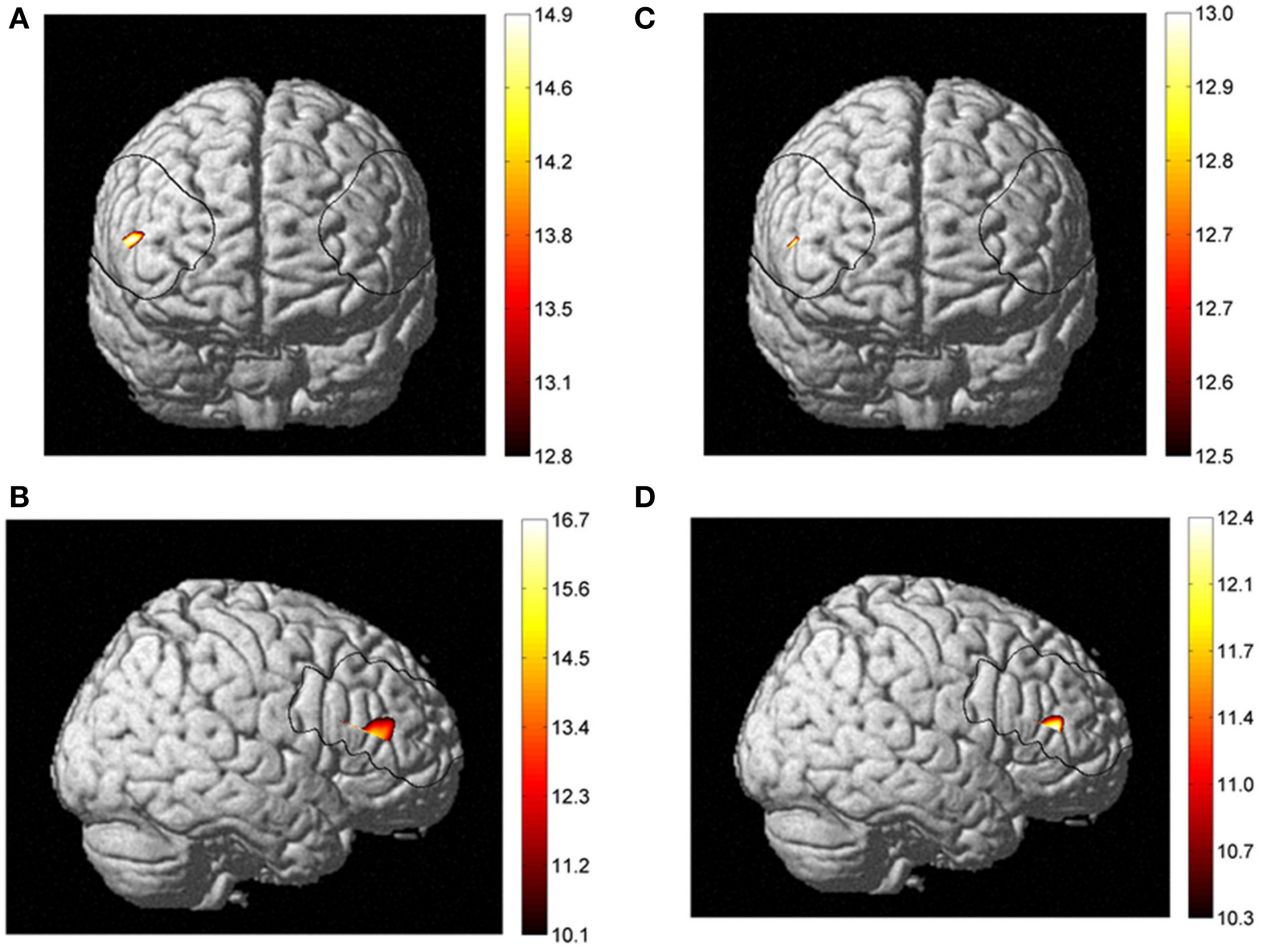
**Fitness effect between higher fit and lower fit women for HbO_2_ in frontal (A) and right sagittal view (B), and for HbT in frontal (C) and right sagittal view (D)**.

Concerning the NIRS data, the ANOVA indicated main effect of Fitness. *Post-hoc* comparisons revealed that on channel 24, which is localized in the right inferior frontal gyrus of the ventrolateral prefrontal cortex, the higher fit individuals had larger changes in cerebral oxygenation (for HbO, [*F*_(1, 54)_ = 7.2, *p* < 0.01 and *F*_(1, 54)_ = 8.4, *p* < 0.01, in naming and executive condition respectively]; and HbT [*F*_(1, 54)_ = 8.2, *p* < 0.01 and *F*_(1, 54)_ = 7.5, *p* < 0.01, in naming and executive condition respectively]) than lower fit individuals on both conditions of the Computerized Stroop task. These results, depicted in the Figure 5 suggest that higher fit had more activation in the right inferior frontal gyrus when performing the Stroop task. We found no effect of age and condition in the NIRS data. All NIRS results (concentration changes) are presented in Table 3.

**Table 3:** **Means and Standard deviations of cerebral changes (Δ) from baseline during both conditions of the Computerized Stroop task**.

	**Lower fit**			**Higher fit**			**Fitness ES (d)**		
	**ΔHbT (A.U)**	**ΔHbO_2_ (A.U)**	**ΔHbr (A.U)**	**ΔHbT (A.U)**	**ΔHbO_2_ (A.U)**	**ΔHbr (A.U)**	**ΔHbT**	**ΔHbO_2_**	**ΔHbr**
Naming condition	−0.93 ± 1.83	−1.29 ± 2.08	0.92 ±1.78	0.57 ± 1.63^*^	0.49 ± 1.81^*^	0.16 ± 1.32	0.9	0.9	0.5
Executive condition	−1.28 ± 1.86	−1.56 ± 2.25	0.93 ± 3.09	0.68 ± 2.10^*^	0.54 ± 2.33^*^	−0.15 ± 2.45	1.0	0.9	0.4

*Results are presented mean ± SD; AU, arbitrary unit*.

* *Different from lower fit p < 0.05; ΔHbO_2_, changes in oxyhemoglobin concentrations; ΔHbr, changes in deoxyhemoglobin concentrations; ΔHbT, changes in total hemoglobin. ES (d), Cohen's d (Effect Size)*.

## Discussion

The aim of this study was to assess the association between cardiorespiratory fitness level, cerebral oxygenation and cognitive performances in younger and older women. Based on the existing literature, we hypothesized that the cardiorespiratory fitness level would selectively enhance executive functioning in a computerized Stroop task. Secondly, we hypothesized that better executive control in higher fit people would be related to increased cerebral oxygenation. The results of this study supported our first hypothesis, as we found that regardless of age, individuals with a higher $\dot{\text{V}}$O_2*max*_, perform better in the executive condition of the computerized Stroop task than individuals with a lower fitness level (lower $\dot{\text{V}}$O_2*max*_). Additionally, this effect was specific to switching (executive) conditions and did not emerge in naming (non-executive) conditions. For our second hypothesis, we found a greater amplitude response in cerebral oxygenation evoked during the Stroop task, in women with higher fitness levels than in women with lower fitness levels. Our results highlight the right inferior frontal gyrus as being selectively more activated in higher fit women in comparison to lower fit women, an effect that was independent of age group.

Regarding our cognitive performance results, the interaction age by condition in the trail test, the paper pencil version of Stroop and the computerized Stroop confirm that the age is accompanied by a decline in cognitive function and more specifically in the executive domain. Also our findings are in accordance with reviews (Hall et al., 2001; Bherer et al., 2013), a meta-analysis (Colcombe and Kramer, 2003) and recent longitudinal and cross sectional studies (Boucard et al., 2012; Predovan et al., 2012) that have demonstrated that older adults with higher fitness levels show specific physical training benefits in the executive function domain. Our findings are in line with, Boucard et al. (2012), who found in older people, a specific effect of cardiorespiratory fitness level only in inhibition (executive condition) and not in non-executive conditions. Similarly, our results align with, Predovan et al. (2012) who reported a specific aerobic training effect in the switching condition of the Stroop task after only 3 months of training in older adults. In addition, consistent with our results, these authors do not report a significant effect of aerobic training in the non-executive conditions of the Stroop task. All these results suggest that cardiorespiratory fitness has a specific positive impact on executive function. It is important to note that new evidence suggests that general improvements in fitness can also selectively improve executive functions. Indeed, Smiley-Oyen et al. (2008) found that aerobic training and resistance training selectively improve the executive functions of older adults. Our results support a positive association between cardiorespiratory fitness level and executive function performance and that the neurophysiological adaptations in the brain regions implicated in executive control are likely at the root of this selective improvement. Although, the anterior cingulate cortex is implicated in response conflict, prefrontal cortex structures contribute to a large range of high-level cognitive functions. Our findings support the proposal that higher cardiovascular fitness might improve functioning in prefrontal brain regions which are sensitive to age-related changes. Indeed, while the aging process is accompanied by brain atrophy that tends to be greatest in the frontal lobe, this decline can be moderated by fitness level (Yuki et al., 2012).

Of the research studies that have reported better cognitive functioning is associated with higher cardiorespiratory fitness levels; those that examined brain activity during cognitive performance have typically found that this is accompanied by an increased activation in the brain. For example, Colcombe et al. (2004) found a higher activation in different frontal and parietal regions during a Flanker task for higher fit older adults as compared to sedentary control people. Also, Rosano et al. (2010) reported that after 12 months of aerobic training, there was a higher activation in dorsolateral prefrontal cortex during the Digit symbol substitution test. In a cross-sectional study, Prakash et al. (2011) also found greater blood oxygen level dependent (BOLD) signal increases in the prefrontal and the parietal cortex during the executive Stroop conditions for older people who demonstrated a higher $\dot{\text{V}}$O_2*max*_. Taken together, these results suggest that a higher cardiorespiratory fitness level is related to higher task-evoked hemodynamic responses in frontal, parietal and temporal areas and better cognitive functioning. Our results are in accordance with these findings since we found, regardless of age, greater response amplitude in the ΔHbO and ΔHbT NIRS signal for higher fit in comparison to lower fit individuals, during the Stroop task. In this study, we found negative value for ΔHbO and ΔHbT in both Stroop conditions for the lower fit subjects. Since, the NIRS signal is a change score, the negative value means a decrease of cerebral oxygenation during cognitive task from baseline (rest). So, the lower fit individuals may not have had enough oxygenated blood to meet the demand during task performance, they may generally have lower resting values, or their blood flow may not be as efficient as higher fit individuals. A decrease of cerebral oxygenation could be due to an important vasoconstriction of cerebral blood vessel. According to recent review (Huang et al., 2014), it's well known that the lower fit individuals presented higher stress (i.e., sympathetic activity) response during cognitive task compared to higher fit individuals. This physiological phenomenon could be involved in the decrease of cerebral oxygenation. However, within the current study, we do not have all the measures necessary to tease-out why the lower fit individuals have negative values and future research is needed to clarify this result. Furthermore, we need to keep in mind that the brain hemodynamic changes associated with fitness level are not systematically manifested by a higher functional activation signal but can in some instances also lead to a decrease in hemodynamic response in certain brain regions and during certain cognitive tasks (Brehmer et al., 2014). In some studies, the decreases in cerebral activity actually suggest a more efficient brain processing in those that are higher fit in comparison to those that are lower fit (Brehmer et al., 2014). However, our results seem to support that higher physical activity levels and greater cardiorespiratory fitness are associated with greater activity in prefrontal regions and better cognitive performance.

Several neurophysiological adaptations inherent to physical activity level, could explain these functional activation changes. Previous reports suggest that better cognitive performance in individuals with higher fitness level are associated with an increase in gray (Colcombe et al., 2003; Weinstein et al., 2012; Yuki et al., 2012) and white matter volume (Colcombe et al., 2003; Johnson et al., 2012; Tseng et al., 2013) and an increased hippocampal volume (Chaddock et al., 2010; Erickson et al., 2011). Weinstein et al. (2012) and Yuki et al. (2012) found that active people had a greater gray matter volume in pre-frontal lobe than less active people. In addition to the potential fitness benefits to the size and structures of the brain, fitness related changes in the vascular health of the brain could also explain our results. Indeed, Davenport et al. (2012), put forward the hypothesis that the cerebrovascular reserve could be the link between cardiorespiratory fitness level and cognition. Certainly, it is well known that higher fit people demonstrate a greater cerebrovascular health and better compliance in endothelial function (Bolduc et al., 2013). Using neuroimaging measures several researchers (Bailey et al., 2013; Murrell et al., 2013) have found that higher fit people demonstrate a higher cerebral blood flow at rest and during exercise. Recently, using MRI techniques, Xu et al. (2014) found that the cerebrovascular perfusion is improved by strength training. However, none of these studies evaluated the impact of improved vascular health on cognition. To support that greater cerebrovascular perfusion and oxygenation is associated with improved cognitive performance, Pereira et al. (2007) reported that after 3 months aerobic training with middle-aged adults, cerebral blood volume in the dentate gyrus of the hippocampus was increased and associated with improved $\dot{\text{V}}$O_2*max*_, suggesting better vascularization. This better vascularization was in line with improvements in declarative memory performance. More recently, Brown et al. (2010), observe that physically women had a greater cerebrovascular responsiveness during exercise in hypercapnia than sedentary women and this responsiveness was correlated to better cognitive functioning. The greater cerebrovascular reserve and vascular compliance is probably mediated by an angiogenesis induced by liberation of neurotrophic factors such as BDNF, insulin-like growth factor (IGF) or vascular endothelial growth factor (VEGF). Also, it's well known that cardiorespiratory fitness improves vasoreactivity and improves endothelial function leading to better perfusion and better brain functioning (Bolduc et al., 2013; Voelcker-Rehage and Niemann, 2013). Our results are in accordance with all these previous findings and more specifically with recent research by Fabiani et al. (2014) that measured cerebral oxygenation with NIRS technique and found that higher fit older adults have a greater response in HbO_2_ during visual task. The current findings support the hypothesis that neurovascular coupling is impaired in low-fit older adults. Our results are consistent with a decreased vascular reactivity in lower-fit older adults. A possible explanation for this is that the vascular system may have lost some of its capacity to adapt to stimuli demands. Also Fabiani et al. (2014), put forward the hypothesis that the lower fit older adults may suffer from decreased cerebral capillary density. This is consistent with the notion that aerobic exercise leads to an increase in angiogenesis, and thus increased perfusion, and that this can lead to improved executive function (Davenport et al., 2012; Fabiani et al., 2014).

Interestingly, our contrast analysis between higher and lower fit individuals revealed a posteriori that the right inferior frontal gyrus was more perfused during both conditions of our computerized Stoop task. This result is in accordance with previous reports using other neuroimaging techniques and highlights the possible implication of this region of interest in better cognitive functioning for higher fit individuals. Colcombe et al. (2004) found that frontal and prefrontal cortex structures showed greater hemodynamic signal increases during a Flanker task (i.e., inhibition task). These authors reported that the right middle frontal gyrus showed greater signal increases for physically active older people. The current finding supports a greater involvement of right prefrontal cortex structures in executive tasks in higher fit individuals. In line with these findings, Voelcker-Rehage et al. (2010) found that the functioning of the right inferior frontal gyrus was modified by physical fitness level during a Flanker task (i.e., inhibition task) and that it could be implicated to better cognitive functioning in highly physically active people. More recently Weinstein et al. (2012), found that the association between aerobic fitness and executive function may be partly mediated by prefrontal cortex volume. More specifically, volume of the right inferior frontal gyrus mediated the relationship between cardiorespiratory fitness and Stroop interference. We would propose that a greater volume in gray matter is associated to better perfusion and this may explain why we found a greater HbO and HbT signals during cognitive tasks in higher fit subjects. In cognitive domain, the right inferior frontal gyrus (rIFG) is one of region of interest (ROI) largely implicated in the executive function and inhibitory control (Aron et al., 2004, 2014; Forstmann et al., 2008). We found that this ROI was more activated in both non-executive and executive conditions. One of the possible explanations is that the rIFG is typically involved in inhibitory control and during conditions requiring attentional control. Hampshire et al. (2010) found that rIFG is recruited when important cues are detected, regardless of whether that detection is followed by the inhibition of a motor response, the generation of a motor response, or no external response at all. However, the role of the rIFG in improved executive functioning in higher fit individuals needs to be interpreted with caution and future research is necessary to confirm its role and sensitivity to physical training.

## Conclusion

In conclusion, this study supports the positive association between a higher cardiorespiratory fitness level and cognitive performance in younger and older women. The results indicate that regardless age, the higher fit individuals performed better in executive conditions than lower fit individuals. These results support previous findings that demonstrate that the executive domain is most sensitive to physical fitness level and that this relationship is likely mediated by neurophysiological changes in prefrontal cortex. Indeed, we did find that higher fit individuals demonstrated an increased task-induced oxygenation response in the right inferior gyrus during both conditions of Stroop task in comparison to lower fit individuals. Improved performance and oxygenation response suggest that independent of age, higher fitness levels can lead to both physiological and behavioral benefits.

### Conflict of interest statement

The authors declare that the research was conducted in the absence of any commercial or financial relationships that could be construed as a potential conflict of interest.
